# Impact of peer-trainer leadership style on uptake of a peer led educational outreach intervention to improve tuberculosis care and outcomes in Malawi: a qualitative study

**DOI:** 10.1186/s12913-020-05386-0

**Published:** 2020-06-05

**Authors:** L. M. Puchalski Ritchie, H. Mundeva, Monique van Lettow, S. E. Straus, E. Kip, A. Makwakwa

**Affiliations:** 1 Department of Medicine, University of Toronto, Toronto, Canada; 2 Knowledge Translation Program, Li Ka Shing Knowledge Institute, St. Michaels Hospital, 30 Bond Street, Toronto, ON M5B 1W8 Canada; 3 Department of Emergency Medicine, University Health Network, Toronto, Canada; 4 Institute of Health Policy, Management and Evaluation, University of Toronto, Toronto, Canada; 5 Dignitas International, Zomba, Malawi; 6 Dalla Lana School of Public Health, University of Toronto, Toronto, Canada; 7 National TB Program, Ministry of Health, PO Box 30377, Lilongwe, Malawi

**Keywords:** Lay health workers, Leadership, Implementation

## Abstract

**Background:**

Little is known about how to build leadership capacity to support implementation of evidence-based practices within health systems. We observed substantial variability across sites in uptake and sustainability of a peer-led educational outreach intervention for lay health workers (LHWs) providing tuberculosis care in Malawi. Feedback from peer-trainers (PTs) suggested that leadership may have contributed to the variation. We sought to assess the impact of PT leadership style on implementation, and to identify leadership traits of more successful PTs, to inform future implementation planning and to identify targets for leadership capacity building.

**Methods:**

Qualitative study employing interviews with PTs and LHWs at high and low implementation sites, and review of study team and quarterly PT meeting notes. High implementation sites achieved high uptake, sustainability and fidelity of implementation including: close adherence to training content and process, high levels of coverage (training most or all eligible LHWs at their site), and outcomes were achieved with high levels of self reported competence with the intervention among both PTs and LHWs. Low implementation sites achieved limited coverage (<= 50% of LHWs trained), and intervention fidelity.

**Results:**

Eight PTs and 10 LHWs from eight high and 10 low implementation sites participated in interviews. Leadership traits of more successful PTs included: flexibility in their approach to training, role modeling and provision of supportive supervision to support learning; addressing challenges proactively and as they occurred; collaborative planning; knowledgeable; and availability to support implementation. Traits unique to less successful PTs included: a poor attitude toward their role as PT and a passive-avoidant approach to challenges.

**Conclusion:**

This study identified leadership traits more common among unit level leaders at sites with higher uptake, sustainability, and fidelity of implementation. These findings provide a starting point for development and evaluation of a leadership capacity building intervention for unit level leaders to support implementation.

## Background

Leadership impacts implementation of evidence-based practice [1–3], with unit level leadership style shown to impact: implementation climate [1, 4], sustainability [5], engagement of providers [6], implementation process outcomes including adoption, penetration, and fidelity [7], and implementation outcomes [2, 6, 7]. While unit level supervisors may impact implementation indirectly through participation in development of policies and procedures, their most commonly discussed areas of impact are likely through direct means, as a result of frequent and close interpersonal contact with frontline staff [8], and may include inspiring and motivating staff, providing support directly, and creating supportive team and unit climate environments for implementation (3,4). As noted by Aaron et al. [9] unit level leaders who directly supervise health care providers may be particularly important in facilitating implementation of evidence-based practices, and note that unit level leaders who do not support new practices initiated by higher level supervisors may impede implementation. A systematic review by Gifford et al. [3] supports this view, finding lack of support from managers (unit level leaders) and other staff to be an important barrier to nurse’s use of research. Additionally, Shuman et al. (4) found nurse managers significantly related to unit climate for the evidence-based practice implementation, primarily through their leadership behaviours.

While a number of leadership styles have been identified as positively (transformational, empowering, approaches which engage staff and set structured expectations) [2, 6, 7] and negatively impacting implementation (passive-avoidant) [2], relatively little is known about how to build leadership capacity to support implementation of evidence-based practices [10]. As noted by Gifford et al. [11] the mechanisms by which leadership impacts implementation are not well understood with identification and understanding of aspects of leadership critical to implementation success essential to development of interventions to build implementation leadership capacity. However, two recent studies found interventions designed to develop leadership capacity, were feasible, acceptable, and perceived as useful [5, 10], and provide a starting point for intervention development.

As the vast majority of studies to date have been conducted in high income country health care settings; less is known about successful leadership styles to support implementation of evidence-based practice in low- and middle-income country (LMIC) settings where lay health workers (LHWs) often represent a substantial proportion of the health workforce.

While lack of and/or poor leadership is known to negatively impact implementation efforts in any setting, LMICs may be particularly challenged to provide strong leadership given the substantial shortage of skilled human resources for health facing such settings both at the level of front line care providers [12] from which unit level leaders are typically drawn and system level leaders [13]. Lack of leadership has been identified as a barrier to implementation of health and health system interventions in LMICs [14–16], with training of leaders for scale-up a suggested approach to address this issue [14]. For example, Lazzarini et al.’s (9) systematic review of barriers and facilitators to effective implementation of maternal near miss case reviews, identified lack of leadership including lack of coordination, monitoring and supervision; lack of understanding of the evidence-based practice process and goals; and lack of involvement of leaders, as barriers to implementation. Yamey et al.’s (10) qualitative study of academic leaders in implementation science with experience in large scale implementation of evidence-based health tools and interventions in LMICs, noted lack of leadership at both local and national levels as important barriers to implementation, and call for research to identify and foster public health leadership. While these studies highlight the importance of good leadership for effective implementation in LMIC settings, traits and behaviours of effective leaders and/or specific approaches to improving leadership are not identified.

During the course of implementation of a previously piloted implementation intervention designed to address LHW training and supervision needs toward improving TB care and outcomes, we observed substantial variability in intervention uptake across sites. Interactions with and feedback from PTs during quarterly meetings suggested that PT approach to leadership may have played a role in uptake. Therefore we sought to assess the impact of PT leadership style on uptake and sustainability of the intervention, and to identify leadership traits of more successful PTs, to inform future implementation planning and to identify targets for leadership capacity building.

### Implementation study

#### Design

The full methods of the implementation study were previously published and are presented briefly here [17]. A pragmatic cluster randomized controlled trial and process evaluation employing qualitative methods were conducted to assess the effectiveness of a refined version of a previously piloted intervention [18] designed to improve provision of care and Tuberculosis (TB) treatment outcomes and to understand barriers and facilitators to scale-up and sustainability of the intervention.

#### Setting

Although improving, TB remains an important public health issue in Malawi [19]. At the start of the implementation study, TB treatment completion rates were 81 and 76% for new and previously treated TB cases respectively [20], remaining below the national target rate of 95% [21]. In Malawi, outpatient TB care is principally provided by LHWs. LHWs are a cadre of paid health workers, who provide health promotion, prevention and a limited number of curative tasks, in the community and local health centres [22]. At the health centre level TB care is provided by LHWs supervised by TB-focus LHWS. TB-focus LHWs are general LHWs who receive up to 2 weeks of additional training specific to TB surveillance, diagnosis and treatment, and are responsible for provision of TB care at their health centre. As the primary providers of outpatient TB care in Malawi, LHWs play a critical role in efforts to achieve treatment completion targets and through this to reducing TB transmission, morbidity and mortality.

### Implementation study intervention

The intervention was based on formative work conducted by our group in which LHWs identified lack of TB knowledge and job skills as the primary barriers to their role as TB care providers [23]. The multi-component intervention employed onsite PT led educational outreach, a point-of-care reminder tool, and a peer support network.

The educational outreach component of the intervention was developed to address this knowledge gap, by providing TB disease knowledge and patient counselling skills, designed to address risk factors for non-adherence based on a review of international and local literature. The point-of-care tool was designed to function as a reminder tool to be used during patient interactions. One side of the tool used simple pictorials to show the progress of a patient through TB treatment, to support patient education and adherence counselling. The other side provided suggested questions to assessing and addressing treatment issues and adherence. Based on feedback in the pilot study, a drug dosing chart was also added for easy reference during patient encounters. Also based on our experience in the pilot study, a small phone credit stipend was provided quarterly to PTs to facilitate development of a peer support network.

### Selection of participants & health facilities

In consultation with the national TB control program TB-focus LHWs were selected as PTs in both the pilot and current implementation study. Given their leadership role as unit-level supervisors in providing training and supervision to LHWS providing TB care at the health centre level, the role of PT was felt most compatible with this cadre of workers, and most appropriate in considering scale-up and sustainability of the program over time.

The implementation study was conducted in collaboration with Dignitas International and included 4 districts in the South East Zone of Malawi, with all health centres routinely providing TB care included in the trial. TB treatment completion rates among participating districts ranged from 69 to 80% at the start of the study [24]. Dignitas International is an academic non-governmental organization (NGO), and was providing support and mentorship to frontline clinical staff and conducting research in the study districts, and provided mentorship to PTs during site visits to participating health centres for regular program activities. The pilot district was excluded from the present study, and a sixth district receiving support from Dignitas International at the time of the study declined to participate.

### Training

Letters were sent to health centres randomized to the intervention group, with a brief description of the program, and asking that the TB-focus LHW be sent to the PT training session. Peer-trainer (PT) training was provided off-site over 1 week, by LPR who also provided the PT training in the pilot implementation study. In line translation was provided as needed by one or more Malawian colleagues’ depending on group size. Peer-trainer training covered content of the cascade training program and practice with the point-of-care tool, as well as, introduction to the approach to training. Approaches to supportive supervision were also briefly introduced, but no formal leadership training was provided. Incentives were not provided for participation in PT training. PTs were then asked to provide cascade training at their health centre with all LHWs routinely involved in TB care at intervention health centres invited but not required to participate in cascade training. Training was provided onsite during regular work hours. Incentives were not provided to LHWs for participation in cascade training.

### Leadership study

#### Rationale

During the course of the implementation study we observed substantial of variability in intervention uptake across sites. Variability in intervention uptake was noted in several areas. First, proportion of LHWs completing cascade training varied substantially, ranging from 0 to 100%. Second, approach and adherence to the cascade training program in terms of both content and process varied. Some PTs provided only a brief introduction to the program or condensed incomplete training; other PTs completed the training program in full as intended; and other PTs provided additional and/or make up sessions for LHWs who missed sessions, to ensure training was delivered in full. Finally, self reported comfort and confidence with use of the intervention varied across low and high implementation sites.

Interactions with and feedback from PTs during quarterly meetings suggested that approach to leadership may have played a role in uptake. Therefore we sought to assess the impact of PT leadership style on uptake and sustainability of the intervention, and to identify leadership traits of more successful PTs, to inform future implementation planning and to identify targets for leadership capacity building.

## Methods

### Design

Multi-component qualitative study employing interviews with PT and LHWs at high and low implementation sites and document review. Documents reviewed included notes from quarterly peer-trainer meetings where issues with and approaches to LHW participation in the training were routinely discussed and study team notes collected as part of the process evaluation which included notes from observations and interactions with PTs and LHWs during field visits by study team members and Dignitas mentors.

### Setting, health Centre and interview participant selection

The study was conducted in all 4 districts in the South East Zone of Malawi participating in the implementation study [17, 18]. Health centres were chosen for participation in the present study from among intervention sites using extreme case sampling [25], with the highest, and lowest implementation health centres within each district chosen to participate. Extreme case sampling is commonly used when some information about program variation is known, to gain an understanding of factors and/or circumstances that contribute to high and low program performance, with the goal of applying lessons learned to improving overall program performance [25].

High implementation health centres were led by more successful PTs, and defined as sites where PTs trained a large proportion of LHWs routinely providing TB care, achieved high fidelity implementation, and achieved both high levels of initial uptake and sustained implementation over the 1 year trial period. High fidelity implementation sites, adhered closely to the training content and process including frequency and duration of training sessions, achieved high levels of coverage completing training with most or all eligible LHWs (all achieved 100% coverage at the start of implementation and 2 trained additional staff that transferred into the site), and both PTs and LHWs reported high levels of competence with the intervention.

Low implementation sites, were defined as health centres where PTs trained only a small proportion (0% to <= 50%) of LHWs routinely providing TB care, and where intervention fidelity was low. In particular, intervention fidelity was defined as low coverage, with less than half eligible LHWs participating in training. In addition, while adherence to both training content and process was variable among low implementation HCs. Adherence was generally lower, with some sites receiving only a brief summary of the intervention and others providing reduced content, frequency and/or duration of training. In addition, LHWs at low implementation sites, generally reported low levels of comfort and competence with the intervention.

Peer-trainers from selected health centres were invited to participate by a trained research assistant, in person or by phone. A list of LHWs providing TB care at selected health centres was compiled by the PTs. LHWs were selected from this list to represent the range of LHW characteristics in terms of gender, age, and years of experience, within the limitations of availability of LHWs at selected sites.

The study and research assistant (RA) were introduced to the LHWs by the PTs. LHWs were then approached in person or by telephone if the selected LHW was not present on site at the time of the health centre visit by the study RA, and invited to participate. Informed written consent was obtained from all participants. Participants received a refreshment and cash stipend equivalent to one USD for their participation.

### Data collection

Interviews were conducted between November 2017 and February 2018, concurrent with collection of trial outcome data. In-person, semi-structured Interviews were conducted by a trained RA, native to Malawi, and fluent in English and Chichewa. Demographic information, including age, years experience working as a LHW, and years of experience providing TB care, was collected at the start of each interview. The interview guide was based on Aarons et al.’s implementation leadership scale, a measure of unit level leadership [1] and PT feedback that was obtained during quarterly meetings (Additional File 1). Areas of interest included: how the program was introduced, the approach employed to teach/engage/support LHW participants, and how challenges to implementation were addressed.

Interviews were conducted in a private location, at or near the participants’ health centre, at a time convenient to participants. Interviews were conducted in Chichewa, digitally audio recorded, and transcribed verbatim and translated by the RA who conducted the interview. To ensure accuracy and conceptual equivalence, all transcripts were verified by a second socio-linguistic translator [26].

Documents reviewed included notes from quarterly peer-trainer meetings where issues with and approaches to LHW participation in the training were routinely discussed. Quarterly meetings were held in English with inline translation provided by the RA and study co-ordinator. Meeting notes were taken independently by two and in some cases three study team members. An initial meeting report was compiled from the hand written notes by the study RA, was circulated to the study co-ordinator and principal investigator, for comment and revision as necessary.

Study team notes were collected as part of the trail’s process evaluation and included notes from observations and interactions with PTs and LHWs during field visits by study team members and/or Dignitas mentors (Dignitas International is an academic NGO operating in the area; Dignitas mentors engaged with PTs at intervention sites during field visits conducted to provide support and mentorship to front line clinical staff).

### Analysis

Interviews were analysed using directed content analysis [27], with interviews as the unit of analysis. NVivo 10 (QSR International Inc., Southport, UK) was utilized to organize and code the data. An initial coding framework was developed based on Aaron et al.’s implementation leadership scale [1] and feedback from PTs during quarterly meetings. Analysis occurred in two rounds. First, two study team members (LPR, HM) read and coded the transcripts independently. The coding framework was then revised through discussion and input from the Malawi based study team members as needed. The revised coding framework was then applied independently by the same two study team members (LPR, HM) with discrepancies resolved through consensus. Themes were sought across individuals with consideration of PT gender, age and years of experience, as well as, district and high and low intervention uptake sites within and across districts.

Meeting notes were reviewed and discussed after each quarterly meeting, with informal notes made of emerging themes. Study team notes were reviewed on a regular basis and notes made of ongoing and/or emerging themes throughout the implementation period.

Methods, data source, and analyst triangulation [25] were employed with interviews, quarterly PT meeting and study team meeting notes. Analyst triangulation involves comparing and contrasting findings generated by different individuals who analyse the data. Methods triangulation involves comparing and contrasting data generated using different data collection methods such as data collected through interviews, observations, and document reviews. Data source triangulation involves comparing and contrasting data from different sources, such as individual interviews, within a data collection method. Convergence and divergence in themes and sub-themes was sought across methods and data sources by each analyst individually, and then across analysts collaboratively. With findings from all sources considered together to provide a comprehensive understanding of how leadership style may influence participation and inform future implementation planning, and to assess sustainability and scalability of the program.

## Results

### Characteristics of participants

Eight PTs and 10 LHWs participated in interviews*.* Four PTs came from high implementation health centres and 4 from low participation sites. Four LHWs came from high implementation sites and six from low implementation sites. PT participants ranged in age from 32 to 53 years, and from 10 to 23 years experience working as a LHW and one to 11 years experience providing TB care. All but one of the PTs were male (7/8). LHW participants ranged in age from 28 to 47 years. LHWs experience working as a general LHW ranged from 9 to 23 years and one to 18 years for providing TB care. Seven of 10 were male.

### Reasons given for choosing to participate or not participate in the cascade training

All LHWs routinely involved in provision of TB care at intervention sites were eligible to participate in cascade training. As of the end of the intervention period, PTs reported a total of 169 LHWs completed the cascade training, 152 initially and an additional 17 who had initially declined participation or were transferred into implementation sites after the initial training period who completed training during the implementation period. Based on the initial number of LHWs eligible to participate in cascade training at the study start for sites with final numbers provided, 83 LHWs declined to participate or failed to complete the cascade training. Seven PTs did not attend the final PT meeting and could not be reached to obtain follow up information on the total number of LHWs that completed training. An additional 5 health centres did not have an opportunity to receive cascade training: 3 health centres did not attend the PT training, 1 PT died before he could begin cascade training, and 1 PT reported at the end of PT training that his site no longer provided TB care.

The two common reasons given for choosing to participate in the educational outreach training were to improve personal knowledge and skills, and to improve patient care. Lack of incentives was the primary reason given for not participating. Other reasons for not participating included heavy workload (too busy), not wanting to be trained by a peer or feeling they already know, all reported by a minority of participants.

### Interview findings

#### Leadership traits of more successful peer-trainers

More successful PTs were reported to be flexible in their approach to training described as a willingness to adjust timing of training sessions or provide additional sessions to accommodate personal and or professional obligations of trainees. They were also noted to role model the program approach with patients, and to provide supportive supervision and constructive feedback.

*“when I see that there are three to four trainees I would teach them and I would teach the rest later. I was dividing my time into two because at the time when others are at office others were at outreach program.“ (PT 2 years experience)*

*“When a patient comes he was inviting us to assist (with him) for us to gain more knowledge.” (LHW 1 year experience)*

*“If a patient was not properly assisted, we (PT) were reminding our colleague about the rules of TB adherence.” (PT 7 years experience)*

More successful PTs were proactive and addressed potential challenges and LHW concerns regarding the program at the start of the training period, often during their introduction of the program, and were perseverant through challenges as they arose during implementation.

*“On the incentives part, after I explained to them about the program, they just accepted to be trained even though they were to receive nothing since it will also help them to gain knowledge which may be used in future.” (PT 10 years experience)*

*“When he came he explained that he wants to strengthen TB drug adherence. ....... So when the peer trainer briefed us about this program we felt good about it because we wanted to use that opportunity so that we struggle no more with such a patient (default patients).” (LHW 11years experience)*

*“For me I was just encouraging them that some things may look useless for now but their use is clearly seen later.” (PT 3 years experience)*

While the majority of PTs reported proactively developing a plan for implementation, more successful PTs tended to use a collaborative approach to planning, calling the LHWs’ together to co-create a training plan.

*“Then he called us that if we would be free we should start meeting the following day, then we chose a day to start meetings, then he started explaining to us what he learnt from the training, it was a good approach.” (LHW 5 years experience)*

*“When I came and briefed them about the program, then I asked them, When do you think we can start the program? Then we agreed on a day to start meetings. When that day came they reminded me to start the training. It was not difficult for me to train them because they were willing to learn.* “ *(PT 10 years experience)*

More successful PTs were perceived by LHWs to be knowledgeable about the program. They were perceived to support implementation by making themselves available to address questions or concerns as they arose by reworking their schedule to be onsite during busy TB clinic days and to be available by phone when off-site.

*“ He comes to work daily unlike us we work in shifts, because if a patient comes with a certain problem we may not be able to handle it. We are able to consult him any day and anytime because he is there.” (LHW 11 years experience)*

*“If she is not available when we call her she was responding. ..... If we have a problem concerning TB patient and we call her she would say I will find you right there at the facility and even if we are outside the facility she would say we will solve that problem the following day.” (LHW 8 years experience)*

#### Leadership traits unique to less successful peer-trainers

Traits of less successful PTs were often opposite to those of their more successful peers, and included: lack of flexibility and role modeling, poor planning and an inadequate approach to addressing challenges proactively or during implementation. A poor attitude toward their role as PT emerged as an issue among less successful trainers, with a few instances of LHWs reporting that their PT refused to provide some or all of the training, despite interest from the LHWs at their site.

*“He was supposed to share with us what he learnt from training despite that we come on different days....If there are certain programs thus what he does. He was supposed to do the same with this program.” (LHW 16 years experience)*

Passive-avoidant behavior was noted among less successful PTs. Some PTs who anticipated lack of incentives to be an issue among potential trainees at their sites, were noted to avoid discussion of incentives when introducing the program, and in some cases created the barrier themselves where LHWs reported being willing and interested in receiving the training even if incentives were not to be provided.

*“Aaaah no … We didn’t finish. Mr X. just explained to us in a summary what he learnt from the training. We didn’t have a serious training..... We didn’t even ask about allowances because he is just our fellow LHW. We just wanted him to share with us the knowledge he obtained.” (LHW 11 years experience).*

*“When he was back from the training we knew about it and we were expecting that he will brief us about the program. When he came back after a week he informed the in charge, then after two weeks he explained to us. When we asked him why he didn’t you tell us about the program all this time he said he was afraid to tell us. We were waiting for him to see what he will do about the program but he didn’t do anything about it.” (LHW 9 years experience)*

#### Findings from review of study team and peer-trainer meeting notes

Lack of incentives was a prominent concern during PT training and early implementation. Approaches to addressing the issue were discussed among PT groups during the last day of training with many suggesting that adopting a proactive approach and addressing the issues during program introduction would be the best strategy. In subsequent meetings many PTs reported this had been the approach employed and had found it successful with many potential participants. In addressing the issue of incentives, less successful trainers tended to explain that they had not received an incentive and that participating in training related to providing care to TB patients was part of the LHWs job. In contrast, more successful trainers tended to emphasize the importance of LHWs work with TB patients and the benefits of the training to both the LHWs themselves and to TB patients. More successful PTs, also continued efforts to engage LHWs who initially declined to participate in training throughout the implementation period, and were flexible in their approach offering one-one sessions to make up missed material; in some cases starting a new block of training sessions with LHWs who had not participated in or had not completed the original training. Some of the more successful PTs addressed challenges by engaging support and advice from leadership at their health centre and/or other PTs in their district. In contrast, a few less successful PTs were found to initially deny challenges at their site, reporting difficulties and low levels of implementation only after field visits by Dignitas mentors or the study team revealed issues.

#### Triangulated findings

Themes common among more successful PTs and themes unique to less successful Pts combined from all data sources, are outlined in Table 1.

**Table 1:** Themes common to more successful PTs and themes unique to less successful PTs

Themes Common Among More successful PTs	Description of Theme
Flexible	• More successful PTs reported to be flexible in their approach to training, including a willingness to adjust timing of sessions and/or to provide additional group or individual sessions to accommodate LHWs personal and/or professional schedules
Role Model	• More successful PTs were noted to role model the intervention approach with patients, and in some cases to see patients with trainees early during training in order to model the approach
Supportive Supervision	• More successful PTs noted to provide supportive supervision with constructive feedback provided to individuals and shared with the group to support learning
Proactive • Addressing challenges • Collaborative planning	• More successful PTs were noted to address anticipated challenges at the start of implementation, by providing a detailed introduction to the intervention, and openly discussing challenges and LHW concerns • More successful PTs were reported to plan collaboratively including LHWs at their site in developing a training and implementation plan
Perseverant • Continued efforts to engage LHWs • Engage support and advice to address challenges	• More successful PTs were noted to persevere through challenges as they arose through the course of implementation, this included continuing efforts to engage LHWs who initially declined to participate and offering additional sessions for LHWs who initially declined to participate and for LHWs transferred in after the initial training. • More successful PTs were also noted to engage support and advice from leadership at their health centre and/or other PTs from their district
Knowledgeable	• More successful PTs were reported to be knowledgeable about TB and the intervention, with LHW trainees reported their PT as a resource for addressing questions and issues as they arose
Supportive	• Morse successful PTs were noted to provide supportive supervision both through constructive criticism and sharing learning’s from patient encounters/issues with the LHW TB team, and by making themselves available in person and/or by phone to provide consultation and guidance
**Themes unique to less Successful PTs**	
Poor attitude	• Some less successful PTs were noted to be unwilling to provide some or all the training despite interest from LHWs at their site
Passive-avoidant	• Some less successful PTs were noted to avoid discussion of potential or actual challenges, and to create barriers to training and implementation as a result, others denied facing any implementation challenges until challenges were revealed through study team site visits

Although some less successful PTs were reported to share some of the themes common among more successful PTs, in general, less successful trainers were noted to exhibit the opposite of those of more successful PTs. In particular, less successful PTs were noted to lack flexibility in their approach to training and implementation, were typically not noted to role model the intervention approach to patient care, were noted to exhibit poor planning for training and implementation, and to display an inadequate or avoidant approach to addressing challenges.

Data source triangulation revealed high levels of agreement with respect to traits of more successful PTs. There were no appreciable differences found based on participant age, years of experience, district, and/or high and low uptake sites. Analysis by gender was limited by the relatively low proportion of female participants. A few discrepancies were noted between PTs perceived/reported approach and that of LHWs from their health centre. Specifically, in one instance a LHW reported that a PT had not completed the entire training program with his team, where the PT reported he had. Additionally, a few LHWs noted only limited information provided in the introduction, where as the PTs reported giving a thorough introduction to prospective participants.

Similarly, methods triangulation found high levels of agreement in identifying traits of both more and less successful PTs. Similar to the discrepancy noted above, in a couple of cases (including the instance noted above where the PT had not completed the training), study team/Dignitas mentor field visits, noted discrepancies between PT reports of intervention uptake at their site specifically with respect to number of LHWs trained and/or ongoing use of the program for patient care.

Triangulation across analysts found no notable discrepancies.

## Discussion

We identified a number of traits more common among successful PTs and less common among less successful PTs including: flexibility in approach to training, role modelling the intervention, providing supportive supervision and constructive criticism, a proactive and perseverant approach to addressing challenges, collaborative planning, and were perceived as knowledge and available to support implementation. In addition we identified two leadership traits relatively unique to less successful PTS, specifically, a poor attitude toward their role as a PT and passive-avoidant behaviour toward addressing challenges. Findings of the present study are generally consistent with studies identifying leadership traits associated with implementation success in high income settings, including intervention uptake, fidelity and sustainability at 1 year.

Our results are similar to those of Aaron et al. [2] who found that leadership that is supportive, perseverant in the implementation process and demonstrative of the importance of the evidence-based intervention to health care delivery was associated with sustainability beyond the initial implementation phase. Aaron et, al [2] also found an association between non-sustainment and passive-avoidant leadership, which aligned with our results.

Our results are also similar to the findings of Gifford et al. [7] who found that program leaders who engaged staff and set structured expectations were found to positively affect a number of implementation outcomes, including adoption, penetration and fidelity of implementation.

To our knowledge this is among the first studies to assess the relationship between unit level leadership style and implementation success in a LMIC setting. Moreover, it is also unique in its consideration of an intervention aimed at improving evidence-base practice among LHWs. Based on our findings, development and evaluation of a leadership training program for LHWs providing unit level leadership, is an important consideration both for scale-up of the current program and to inform future implementation initiatives. Although not assessed in the present study, several interactions and events during the course of implementation suggest that the leadership style of higher level leaders, might also have an important impact on implementation, and suggest this as an important area for future research.

### Strengths and limitations

There are several strengths of the present study. Use of extreme case sampling provided an opportunity to compare and contrast the leadership styles of PTs at the most and least successful implementation sites, providing a rich understanding of traits associated with implementation success and identifying potential targets for development of a leadership capacity building intervention to support future implementation efforts in this setting. Use of constructs from a validated tool (the implementation leadership scale) [1], allowed for assessment of leadership traits known to be related to implementation success. While use of qualitative methods, provided an opportunity for the emergence of themes that may be unique to the study setting, given the difference in setting and health worker population participating in this study relative to the general leadership literature. Finally, use of multiple data sources, provided an opportunity to check the accuracy of PT self-reports, and a more in depth understanding of leadership traits related to more and less implementation success.

There are several limitations to the present study. Limitations of the gender distribution of the pool of PTs and LHWs at high and low implementation sites, limited the gender distribution of our interview sample and as a result limited our ability to examine the relationship between gender, leadership style and implementation. Given the potential influence of gender, our results may be less generalizable to settings with higher proportions of female LHWS, and to settings with different work place and/or socio-cultural norms, with future efforts to examine the relationship of gender and leadership needed. Given the relatively small numbers of LHWs at some participating sites, to avoid potential for participants to be identified, linking of PT and trainee interviews was not possible, and results therefore presented in aggregate. As a result of high levels of turnover among district level leadership during our study, we were unable to assess the impact of higher level leadership on implementation. Finally, as this study was conducted in the context of an intervention focused on behaviour change among LHWs and in the Malawi TB program context, findings may not be generalizable to other health worker cadres, countries, or intervention targets.

## Conclusion

This study identified a number of leadership traits more common among unit level leaders at sites with higher uptake and fidelity of implementation, including: flexibility in approach to training, role modelling the intervention, providing supportive supervision and constructive criticism, a proactive and perseverant approach to addressing challenges, collaborative planning, and were perceived as knowledge and available to support implementation. Less successful PTs often displayed opposite traits, such as lack of flexibility and poor planning. A poor attitude toward their role as a PT and passive-avoidant behaviour toward addressing challenges, were relatively unique to less successful PTs. These findings provide a starting point for development of a leadership capacity building intervention for unit level leaders to support implementation activities.

## Supplementary information

**Additional file 1.** Interview Guide.

## Data Availability

Data from this study will not be shared in keeping with the guarantee during the consent process that transcript data would be accessible only to the study team.

## Abbreviations

LHW: Lay Health Worker
LMIC: Low- and Middle-Income Country
PT: Peer-Trainer
RA: Research Assistant
TB: Tuberculosis
