# Supplementary material for: Impact of peer-trainer leadership style on uptake of a peer led educational outreach intervention to improve tuberculosis care and outcomes in Malawi: a qualitative study
Source: BMC Health Serv Res. 2020 Jun 5;20:513. doi: 10.1186/s12913-020-05386-0 (PMC7275352; doi:10.1186/s12913-020-05386-0)
Supplement: Supplementary file 1 — Additional file 1. Interview Guide. [file 12913_2020_5386_MOESM1_ESM.pdf]

Additional File 1: Interview Guide

1. Before we begin, do you have any questions?
    a. Yes (if yes, answer questions)
    b. No (if no, proceed with interview)

2. I would first like to ask a few questions about yourself
    a. Gender: F/M
    b. How old are you?
    c. Are you a HSA?
        i. Yes or no (if no, thank them for their time and terminate the interview)
        ii. If yes, are you a general HSA? TB focus HSA? Or have other special training (microscopy, HTC, etc.)
        iii. If yes, do you routinely provide care to TB patients?
    d. How many years have you worked in this position?
    e. Do you work in a rural or urban health centre?
        i. Clarify as needed: urban = health center within a city or town, rural = health center in a remote location
    f. Have you previously worked as a HSA?
        i. If yes, how many years?
        ii. If yes, how many years have you worked with TB patients?

3. Have you completed the TB/HIV adherence training? (yes/No)
    a. If yes, when?
    b. If yes, how many sessions did you complete? (if less than 8 probe to see if completed all or part of training)

4. What reasons did you/your team give for deciding whether or not to do the training?
    a. For peer trainers, probe for reasons given by both those who did and did not complete the training.

5. How did you/your peer trainer approach the training?
    a. How did you/your peer trainer introduce the program?
    b. How did you/your peer trainer invite participation?
    c. How did you/your peer trainer address concerns over lack of incentives, timing of meetings, etc.?

6. What has been your experience as a peer trainer/participant in the TB/HIV program?
    a. Did you/your peer trainer outline a clear plan on how this TB/HIV program would be run?
    b. Did you feel you/your peer trainer are knowledgeable about TB/HIV adherence program? About TB/HIV care?

        i. Are they able to answer your questions about the training? Tool? and about TB/HIV care?
            1. If yes, do they explain things clearly?
    c. Are they available when you need them?
    d. Did you/your peer trainer recognize the efforts of you/others HSAs who participated in this program?
        i. Do you support your team/ feel supported by your peer trainer in your learning? In the implementation of the program when caring for TB patients?
    e. How did you/your peer trainer approach challenges to implementing the program?
        i. How were problems encountered addressed? Probe for head on, other
        ii. What worked well? What didn’t work well?
    f. What do you think they could do differently to help this program run smoother?
        i. What could they do differently to help you complete your work better?

7. Do you have any advice or suggestions for peer trainers as to how they could better support implementation of the TB/HIV adherence program?

8. Do you have anything else you would like to add?

9. Thank you for your time.
